# Continued poxvirus research: From foe to friend

**DOI:** 10.1371/journal.pbio.3000124

**Published:** 2019-01-30

**Authors:** Susanna R. Bidgood

**Affiliations:** MRC Laboratory for Molecular Cell Biology, University College London, London, United Kingdom

## Abstract

The eradication of smallpox is one of the greatest medical successes in history. Vaccinia virus was made famous by being the virus used in the live vaccine that enabled this feat. Nearly 40 years on from that success, this prototypical poxvirus continues to empower the exploration of fundamental biology and the potential to develop therapeutics against some of the major causes of death and disease in the modern world.

## Abstract 2

The eradication of smallpox was one of the greatest medical successes in history. In this Research Matters article, Susanna Bidgood explores the future of using poxviruses as versatile tools for cell biology research.

In 1980, the World Health Organisation announced an extraordinary scientific achievement that would change the course of human history: their 14-year international vaccination campaign had finally and completely eradicated smallpox. This disease, caused by the poxvirus variola, first appeared around 10,000 B.C. and was the most deadly human disease to ever exist. With a devastating 20% to 60% mortality rate, smallpox killed an estimated 300 million people in the 20th century alone and left its few survivors severely disfigured, with a third of them blind [1].

The practice of vaccination had begun much earlier, in the 18th century. It was commonly recognised at the time that milkmaids, who would catch cowpox from their cattle, never died of smallpox. The English physician Edward Jenner then systematically infected individuals with this nonlethal yet close relative of variola virus and showed that cowpox infection protected a person from smallpox. However, it took the World Health Organisation vaccination campaign, now using a vaccine containing the poxvirus vaccinia, to finally rid the world of the devastating killer [1,2].

Health officials subsequently stopped routine smallpox vaccinations because the risk of using a live virus vaccine against a disease that no longer posed a threat far outweighed the benefits. Due to its bioterrorism potential and likely lethality upon accidental exposure, scientific research on variola virus is now restricted exclusively to government secure facilities where the few remaining stocks of variola virus are kept safely under lock and key. Smallpox has been defeated.

This extraordinary victory was achieved before I was born, so I never received a shot of vaccinia to protect me from smallpox. In fact, for most people alive today, vaccinia virus is simply the vaccine once used to protect people from a disease that no longer exists. Despite this, just four years ago, I established a research project to study the fundamental biology of vaccinia virus.

I was excited to study vaccinia because, independent of its disease-eradicating heroism, this virus is a powerful tool for cell biology research. Vaccinia virus lends itself to experimental manipulation by being both relatively safe, and quick and easy to perform experiments with. It can be handled at room temperature, maintains infectivity after multiple rounds of freeze-thawing, and is able to infect a broad range of species and cell types. The virus naturally undergoes homologous recombination during viral replication, which enables the easy addition or deletion of genes from its genome, and its complete replication cycle only takes 8 to 12 hours. Moreover, as it has been evolving alongside animals for thousands of years and is highly adapted to control the host cell, establish its replicative niche inside the cell, and down-regulate the host’s immune surveillance systems, I can learn a lot of cell biology by watching how it does this.

In recent years, three specific vaccinia features have proved hugely valuable both for the development of therapeutics to treat major public health threats as well as aiding the development of cutting-edge imaging technologies that will be applicable to all areas of cell biology.

Firstly, vaccinia encodes almost all of its own replication machinery despite being an obligate intracellular parasite. Many of these viral proteins are homologues of cellular proteins, giving scientists like me a simpler system in which to begin to understand the function of the closely related cellular proteins. For example, human cells encode over 600 kinase enzymes. As a result, it is sometimes hard to define the role of an individual human kinase within the cell. Vaccinia virus by contrast encodes just two kinases. One of these kinases, called B1, was first identified in the 1980s. The kinase activity of B1 was extensively studied and shown to be essential for viral DNA replication. A decade later, a screen for novel human proteins identified two proteins whose sequences were more than 40% identical to vaccinia B1, suggesting that these proteins would also be active kinases [3]. Today, we know that there is a whole family of human vaccinia-related kinases (VRKs) playing key roles in regulating cellular DNA replication, cell cycle progression, and cell proliferation. Given that cancers can establish themselves when these processes go wrong, VRKs are implicated in several cancers including liver, lung, and breast cancer [4–6]. As a result, this kinase family serves as potential targets for developing cancer therapeutics, with comparative studies of vaccinia B1 still guiding understanding [4,7]

The second key vaccinia feature is its size. Boasting dimensions of 350 nm × 250 nm × 250 nm, vaccinia virus is large (for a virus!) and can be easily tracked by live cell microscopy. In 2008, the power of this feature was exemplified when a novel virus entry pathway into cells was discovered using vaccinia [8]. Jason Mercer, a scientist in Zürich, imaged vaccinia virions as they interacted with the surface of human cells. Upon contact with the plasma membrane, the virus caused the whole cell to produce blebs followed by the internalisation of the virion into a large cellular intrusion. This process was highly reminiscent of the uptake of debris released from dying cells, during a process called ‘apoptotic clearance’. Jason’s further experiments showed that vaccinia displays lipids on its surface, making it look just like a piece of debris shed from a dying cell, and thus vaccinia tricks cells into actively taking it up.

This ‘apoptotic mimicry’ entry pathway is now known to be employed by 20 clinically relevant viruses, including chikungunya virus, dengue virus, hepatitis A virus, Lassa virus, Marburg virus, and Ebola virus [9]. During the 2014–2016 outbreak, Ebola killed over 11,000 people [10]. Since then, the development of anti-Ebola therapeutics to combat future outbreaks has garnered massive investment. Blocking the Ebola apoptotic mimicry entry pathway is a promising approach. These life-saving studies are only possible because 10 years ago Jason spent some time watching how a virus that does not even cause disease in humans, vaccinia, gets into cells. This story illustrates the value of fundamental research aimed at better understanding biology rather than specifically developing therapeutics. Such studies consistently provide new understanding, which later enable the development of clinical therapies. No one in 2008 would have predicted that Jason’s work with vaccinia would prove valuable in combatting Ebola epidemics. Yet performing the experiments with vaccinia was much safer and quicker than performing them with Ebola.

The final key feature of vaccinia is its unusual subviral shape, which can clearly be seen by electron microscopy (Fig 1). Poxviruses house their genome inside a peanut-shaped shell, known as the virus core. Two additional protein structures, known as lateral bodies, sit on the outer concave surface of this shell, and the whole lot is wrapped in a membrane. Although unresolvable by standard light microscopy techniques, when fluorescently tagged, these three subviral structures are resolvable using cutting-edge super-resolution microscopes. Large amounts of stable vaccinia virions can easily be purified and bound directly to coverslips, allowing the simultaneous imaging of hundreds of isolated virions. In these images, the elongated core and flanking lateral bodies provide three-dimensional orientation information, whereas the very small distance between the two lateral bodies challenges the resolution limit of the microscope.

**Fig 1:**
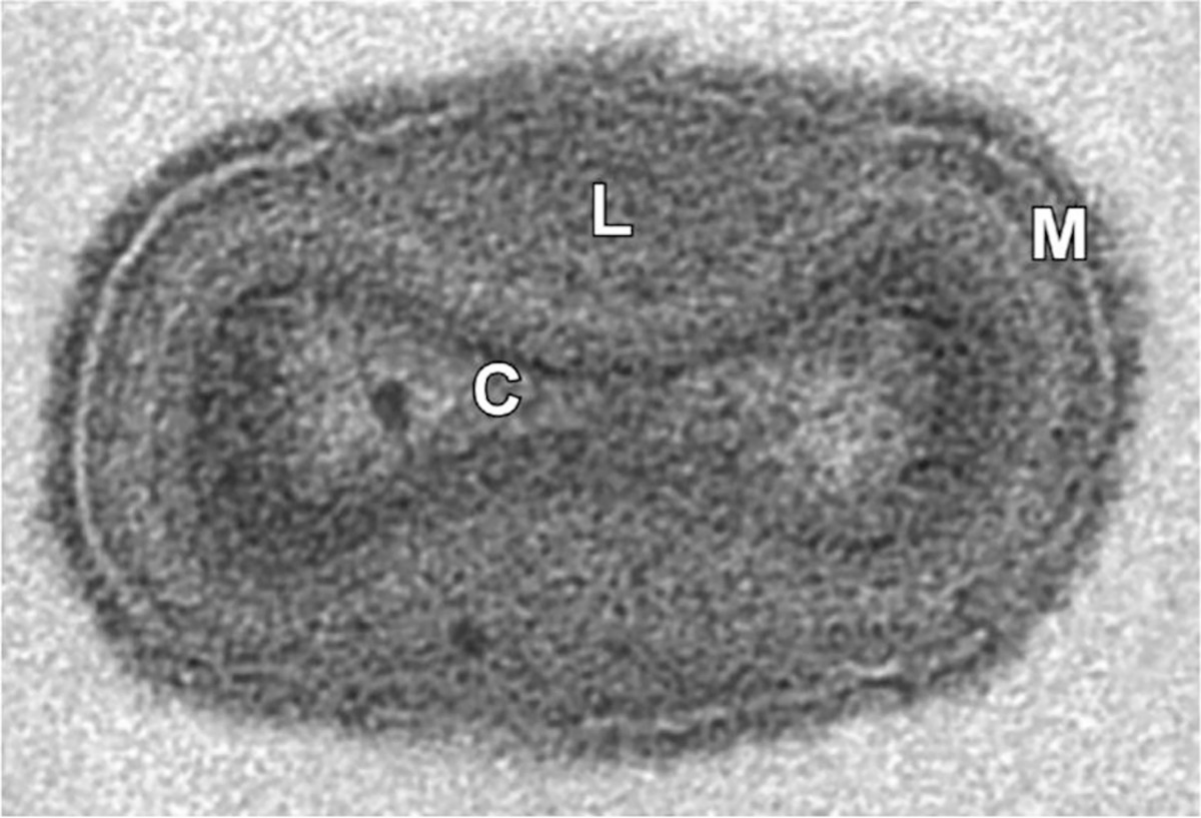
An electron micrograph of vaccinia virus. The three subviral structures, core (C), lateral bodies (L), and membrane (M), are labelled. Reproduced with permission of Jason Mercer.

Two years ago, Rob Gray, a PhD student in London, combined all these features to develop a piece of software that enhances the precision of the super-resolution images. His software automatically detects individual viruses within the image, aligns them, and then averages them together to produce high-confidence models with a 2-fold improvement in resolution. This is a general software that can be applied to a wide array of other data sets independent of the microscope used to collect the images [11].

Vaccinia has also proved to be a powerful tool to enhance the accuracy of super-resolution microscopes. In her paper last year, Siân Culley developed software to automatically detect artifacts sometimes generated during the post-acquisition processing of super-resolution microscopy images. Siân used vaccinia images as test data sets during the development of the software [12]. Their critical size and unusual subviral shape, combined with the plentiful electron microscope images available, made them ideal candidates for testing how well the software was detecting problems in the super-resolution images.

The super-resolution applications to date have employed isolated virions, but it seems likely that soon, similar approaches will be applied to the context of the infected cells. Therefore, vaccinia is proving an excellent tool to aid the improvement of light microscopy approaches. It follows that the better our microscopes, the more new biology we will be able to see and understand.

The eradication of smallpox was undeniably a world-changing accomplishment, but vaccinia virus is more than simply the vaccine that enabled this feat. Vaccinia has a lot more biology to teach us. With vaccinia virus as a tool, poxvirologists all over the world are pushing the boundaries of technology, discovering fundamental biology, and developing therapeutics to combat some of the major causes of death and disease in the modern world.

## Abbreviation

VRK: vaccinia-related kinase
